# Acoustofluidic separation of cells and particles

**DOI:** 10.1038/s41378-019-0064-3

**Published:** 2019-06-03

**Authors:** Mengxi Wu, Adem Ozcelik, Joseph Rufo, Zeyu Wang, Rui Fang, Tony Jun Huang

**Affiliations:** 1 Department of Mechanical Engineering and Material Science, Duke University, Durham, NC 27708 USA; 2 Mechanical Engineering Department, Aydin Adnan Menderes University, 09010 Aydin, Turkey; 3 Department of Molecular and Cellular Biology, Harvard University, Cambridge, MA 02138 USA

**Keywords:** Engineering, Chemistry, Nanoscience and technology

## Abstract

Acoustofluidics, the integration of acoustics and microfluidics, is a rapidly growing research field that is addressing challenges in biology, medicine, chemistry, engineering, and physics. In particular, acoustofluidic separation of biological targets from complex fluids has proven to be a powerful tool due to the label-free, biocompatible, and contact-free nature of the technology. By carefully designing and tuning the applied acoustic field, cells and other bioparticles can be isolated with high yield, purity, and biocompatibility. Recent advances in acoustofluidics, such as the development of automated, point-of-care devices for isolating sub-micron bioparticles, address many of the limitations of conventional separation tools. More importantly, advances in the research lab are quickly being adopted to solve clinical problems. In this review article, we discuss working principles of acoustofluidic separation, compare different approaches of acoustofluidic separation, and provide a synopsis of how it is being applied in both traditional applications, such as blood component separation, cell washing, and fluorescence activated cell sorting, as well as emerging applications, including circulating tumor cell and exosome isolation.

## Acoustofluidics: From lab to clinic

Research into using sound waves to separate biological particles from fluid moving through tiny channels is rapidly advancing, but more is needed to develop real-life clinical applications. Tony Jun Huang of Duke University in the US and colleagues reviewed the latest studies in the rapidly growing field of ‘acoustofluidic separation’. While progress has been made in using the technology to separate micro-sized objects like cells and bacteria from fluids, few breakthroughs have been achieved for separating nanoparticles, like lipids and viruses. Also, advances are needed to speed up the separation process. Still, the field shows much promise for the development of label-free, point-of-care devices for cancer diagnosis, placental health monitoring, and isolating bacteria from water or food. Focusing on product development and technological improvements will enable this technology to find real-world applications, the researchers conclude.

## Introduction

Many advances in medicine over the past century can be attributed to the development of innovative techniques for separating particles and cells of interest from complex mixtures. For example, the development of penicillin, which has saved tens of millions of lives since its debut in World War II, would not have been possible without the efforts of scientists who discovered methods to isolate the drug from culture media^1^. Alexander Fleming, who serendipitously discovered the bacteria that produces penicillin in 1928, is considered by many to be the father of antibiotics. However, after Fleming’s discovery, very few people, including Fleming himself, envisioned practical therapeutic applications of penicillin^2^. There was no efficient way to isolate or characterize the unstable compound, and as a result, research on penicillin was largely halted. Over the next 15 years, improvements in the ability to isolate highly pure penicillin from culture media contributed to the rapid growth in the manufacturability of penicillin in the United States. For example, at the end of 1941, the United States did not have enough penicillin to treat a single patient; by the end of 1943, it was producing enough penicillin to treat the entire Allied Armed Forces^3^. The history of the development of penicillin serves as an excellent example of the importance of separation technologies in bringing biological discoveries to clinical relevance.

In recent years, acoustofluidic^4–11^ (i.e., the fusion of acoustic and microfluidic) separation has increasingly been applied to address many challenges in biomedical research, particularly in the areas of clinical diagnostics and therapeutics^12–15^. Acoustofluidic separation offers a label-free approach that relies on the differential effect of acoustic streaming and radiation forces acting on particles suspended in a liquid^6,8,16–18^. Acoustofluidic systems have been designed to separate particles with different sizes, as well as particles with different physical or mechanical properties^16,19,20^. The applied acoustic waves are typically in a similar frequency and power range as those used in ultrasonic imaging and can be tailored to avoid damage to particles, cells, and organisms. By integrating acoustic manipulation strategies with microfluidic flow paths for liquid handling, miniaturized systems have been developed to isolate, concentrate, and filter bioparticles with the advantages of improved spatial and temporal separation resolution and the potential to be developed into point-of-care diagnostic platforms (due to decreased power and reagent consumption, smaller dimensions, reduced costs, potential disposability, and lower minimum sample volume requirements)^21–23^.

While acoustofluidics offer one approach for separating cells and bioparticles at the microscale, a variety of alternative microfluidic techniques for separation have also been developed. In general, microfluidic cell and particle separation techniques can be categorized into label and label-free methods. Furthermore, based on the separation mechanism, they can be divided into active and passive methods. Methods that utilize applied fields, including magnetic, electrical, optical, and acoustic fields, are referred to active separation methods^24^. Filtration, pinch flow margination, deterministic lateral displacement, and surface affinity based separation are passive methods^25^. Typically, passive methods involve simpler equipment setups; however, active methods have better flexibility and can achieve superior separation resolution due to their ability to exploit differences in mechanical properties, as well as differences in their electric, magnetic, and acoustic properties^26–29^. A comparison of different conventional^30–45^ and microfluidic exosome separation methods^13,46–51^ is given in Table 1 highlighting various parameters for the isolation of exosomes from blood or other biological fluid samples. Overall, Table 1 provides a general trend for how different approaches compare in terms of their yield, purity, biocompatibility, and throughput (flow rate) for a given application.

**Table 1:** Comparison of different exosome separation methods and their separation performance

Methods	Isolation principle	Yield (%)	Purity (%)	Throughput	Advantages	Disadvantages
*Conventional methods*						
Ultracentrifugation^30,31,38–41^	Density and size differences	5–50	23–70	4–12 h	Eligible for processing large volume samples, unbiased isolation	Exosome fusion, soluble protein contamination
Density gradient centrifugation^30,31,42,43^	Density differences	25–50	Not described	8–16 h	Lower levels of contamination from soluble proteins, unbiased isolation	Additional buffer preparation required
Ultrafiltration^30,38,39,42,44^	Size difference	14–35	70–82	2–3 h	Unbiased isolation	Low soluble protein removal rate, exosomal structure damage, protein aggregate
Immuno-magnetic isolation^30–33,39,45^	Antibody capture and magnetic force	13–60	26–78	~3 h	Low soluble protein contamination, eligible for specific exosome subpopulation isolation	Limited availability of robust capture antibodies, additional washing and preparation steps needed, may lose the full functionality of exosomes after elusion
Exo-Quick^30–32,34,35,38,39^	Precipitation	40–80	28–87	~12 h	Unbiased isolation, low structural damage	Contamination from soluble proteins
Field flow fractionation^36,37^	Size difference	Not described	Not described	~24 h	Ability to isolate subsets of exosomes	Small volume samples (100 µL), lengthy procedure
*Microfluidic methods*						
Microfluidic immunoaffinity (ExoChip)^46–48^	Antibody capture	42–94	87–97	8–16 µl/min	Low soluble protein contamination, eligible for specific exosome subpopulation isolation	Limited availability of robust capture antibodies, additional washing and preparation steps needed, may lose the full functionality of exosomes after elusion
Dielectrophoretic (DEP) separation^49^	Size, polarizability, and dielectrophoretic force	Not described	Not described	~30 min	Low soluble protein contamination, unbiased isolation	Potential structural damage
Ciliated micropillars isolation^50^	Size difference	15–60	Not described	~10 min	Low contamination	Only used beads and liposomes for validation
Deterministic lateral displacement (DLD)^51^	Size difference	Not described	Not described	0.1–0.2 nL/min	High exosome integrity	Device prone to clogging, ~60 hour processing time
Acoustofluidics^13^	Size and acoustic contrast factor	~82	~98	4 µL/min	High exosome integrity, unbiased isolation, no requirement of additional reagent and washing steps	Soluble protein contamination

For batch mode processes, throughputs are reported in terms of the total time required to isolate exosomes from the sample. For continuous mode processes, throughputs are reported as volumetric flow rates

Acoustofluidic separation is highly scalable, capable of manipulating bioparticles ranging in size from tens of nanometers to several hundred micrometers. This has important real-world implications because many biological targets, including the biological targets that have been identified for the development of liquid biopsies, also span this same size range. Liquid biopsies are blood-based tests that offer a minimally invasive alternative to traditional tissue biopsies. In addition to the ability to perform early-stage disease diagnostics, liquid biopsies can identify specific genetic mutations, enabling doctors to develop personalized treatments and monitor patient responses. Biological targets identified for liquid biopsies include exosomes (30–150 nm in diameter)^52^ and CTCs (8–20 µm in diameter)^53^. Although the potential value of CTCs and exosomes have been known for over a decade, there has only been one liquid biopsy approved by the U.S. Food and Drug administration (FDA)^54^. This is due in large part to the fact that traditional separation tools, such as centrifugation, are often not capable of isolating these circulating biomarkers with sufficient purity or yield. However, acoustofludic separation is one of the few microfluidic techniques that been used to successfully isolate both exosomes and CTCs from body fluids^13,55^. In addition to isolating circulating biomarkers for diagnostic applications, the versatility and precision offered by acoustofluidic separation techniques have been applied to expand the capabilities of traditional applications, such as fluorescence activated cell sorting^56^, apheresis^57^, and droplet sorting^58^.

Due to these favorable attributes, acoustofluidic separation has been increasingly studied from an engineering perspective and applied to address niche biological problems; however, most of these applications have been limited to academic research rather than solving clinical problems. With this review, we aim to introduce acoustofluidic separation to a wider audience in order to bridge the gap between engineering capabilities and real-world applications. We will present the underlying theory, compare different technologies, and discuss current applications and future potential of acoustofluidic separation.

## Theory and mechanism on acoustofluidic separation

**Fig. 1:**
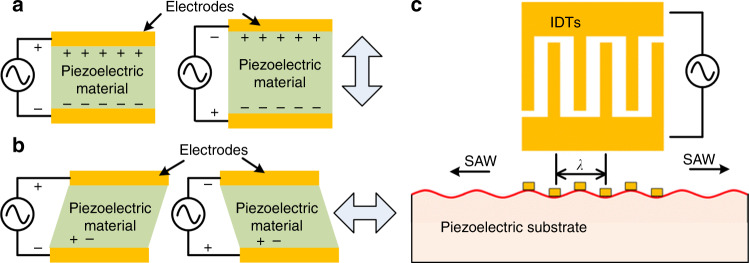
Generating acoustic waves via piezoelectric materials. **a** When a voltage is applied to the electrodes, the piezoelectric material expands and contracts normal to the surface. This mode of vibration is called thickness mode. **b** For some material orientations, when a voltage is applied, the piezoelectric material will deform in the horizontal direction. This mode of vibration is called shear mode. **c** By exciting interdigitated transducers (IDTs) patterned on a piezoelectric crystal, vibrations can be generated on the surface of the material in the form of surface acoustic waves (SAWs). The wavelength of the SAW (*λ*) is dependent on the width and spacing between IDT fingers

Acoustofluidic separation is based on the interaction of acoustic waves with fluids and inclusions within the fluids^6,59,60^. One convenient way to generate acoustic waves is to use transducers made of piezoelectric materials. Piezoelectric materials are able to generate electrical polarization under an applied mechanical stress or, vice versa, mechanical deformation arise from electrical polarization. There are various types of piezoelectric transducers based on their material properties, configurations, and actuation modes^61,62^. Some materials like quartz show natural piezoelectric properties due to its crystal structure yielding a net electrical dipole; while others like lead zirconate titanate can be made piezoelectric by applying an external electric polarization. Depending on the material and orientation, the vibration mode can be different. The most common vibration modes used in piezoelectric transducers are thickness expansion mode and thickness shear mode, as shown in Fig. 1.

When an alternating current (AC) signal is applied to the planer electrodes of a transducer, piezoelectric materials vibrate at the frequency of the AC signal. In both the thickness expansion and thickness shear modes, the whole body of the piezoelectric material vibrates, producing waves that are usually referred as “bulk acoustic waves” (BAW)^63^. An alternative type of vibration, which only happens on the surface of an elastic material, is called a “surface acoustic wave” (SAW). A SAW propagates along the solid-fluid or solid-air interface and decays exponentially into the depth of the solid body^64,65^. SAWs can be generated by using interdigitated transducers (IDTs) patterned on the piezoelectric materials as shown in Fig. 1c. When an AC signal is applied, the IDTs excite the piezoelectric material to generate propagating SAWs. In this case, the resulting frequency, amplitude, and wave-front orientation of the acoustic waves are defined by the dimensions of the electrodes, speed of sound in the material, input power of the applied electrical signal, and the design of the IDTs. For example, the frequency of the acoustic waves is defined by *v*/*λ*, where *v* is the speed of sound in the piezoelectric material and *λ* is the acoustic wavelength. The wavelength (*λ*) of SAW is dependent on the width of the IDTs, as well as the spacing between IDTs as shown in Fig. 1c. There are two types of SAWs: Rayleigh and shear-horizontal SAW. However, the transverse component of the Rayleigh SAW is in the vertical direction and can leak into a fluid domain resting on the propagation direction of acoustic waves, while a shear-horizontal SAW has an in-plane transverse motion that cannot couple with the fluid. Rayleigh waves, which were first studied by Lord Rayleigh in 1885^66^, are used in acoustofluidics extensively for fluid and particle manipulation; while shear-horizontal SAWs are mainly used for sensing applications.

**Fig. 2:**
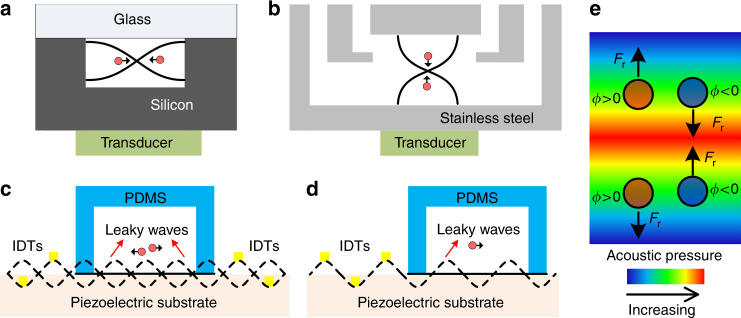
Schematic representation of general acoustofluidic separation techniques. **a** Standing acoustic waves are excited in the half-wavelength resonator formed by a silicon-based microfluidic channel. **b** Microfluidic channels are formed by stainless steel or other high acoustic impedance materials by stacking several layers together. A transducer is attached to the channel to excite vibrations. Standing waves are generated by the reflection of waves at steel/fluid interface. **c** PDMS channels are bonded in between two IDTs. A standing SAW is formed on the piezoelectric substrate by the interference of oppositely propagating SAWs. Upon contact, SAWs leak into the fluid domain in the form of leaky waves. **d** One pair of IDTs generate traveling SAWs that leak into the fluid inside the PDMS microfluidic channel. **e** Particles are directed towards lower (higher) acoustic pressure regions through the effect of acoustic radiation force (Fr) if the acoustic contrast factor (*Φ*) is larger (smaller) than zero

The commonly used types and geometries of the acoustofluidic separation devices are schematically illustrated in Fig. 2. For the BAW-based devices^14,19,67–70^, the microfluidic channel is typically made of materials with high acoustic impedances, such as silicon, glass, or stainless steel. Because of the significant impedance mismatch between the channel material and the fluid medium, the channel walls serve as nearly perfect reflectors for acoustic waves. By tailoring the width or depth of the channel to match half-integer multiples of the acoustic wavelength (Fig. 2a, b), an acoustic resonator is formed^63,71,72^. In the SAW-based devices^73,74^, one can form a standing acoustic wave field by placing two pairs of IDTs to generate SAWs traveling in opposite directions. The interference of two counter-propagating SAWs results in the formation of a standing SAW field in the area between the IDTs. The standing SAW field can be coupled to the fluid domain through a microfluidic channel so that the leaky SAWs excite longitudinal acoustic waves in the liquid, as shown in Fig. 2c. The standing acoustic wave field generated by either SAWs or BAWs forms a distribution of minimum and maximum pressure regions called pressure nodes and antinodes in the fluid domain. Forces generated in this periodic pressure fluctuation are used for particle and cell separations. Besides the standing wave approach, traveling SAWs can also be used to achieve separation^75–82^. In this case, one pair of IDTs generates propagating SAWs perpendicular to the channel, as shown in Fig. 2d. As it will be explained in more detail, differential effects of the traveling SAWs are exploited for separating different cells and particles. A general comparison of different acoustofluidic separation methods is given in Table 2.

**Table 2:** Comparison of acoustofluidic separation methods

Type	Advantages	Disadvantages	Applications
Bulk acoustic waves^14,19,69,70^	Simple device architectures; high throughputs	Difficulties in handling nanoparticles; cooling required due to excessive heat	Platelet separation; plasmapheresis; blood processing
Standing surface acoustic waves^11,71,79,80^	High precision; easy to miniaturize; strong acoustic radiation force	Low throughput	Nanoparticle separation; rare cell separation
Traveling surface acoustic waves^75–82^	High precision; easy to miniaturize; single IDT required	Low throughput; design consideration to prevent standing wave formation	Nanoparticle separation; fluorescence-activated cell sorting

Acoustofluidic separation happens by virtue of an interplay between acoustic radiation forces and acoustic streaming induced drag forces. Any gradient generated in the acoustic field due to interactions such as scattering, absorption, reflection, dampening, or interference of acoustic waves results in acoustic radiation forces and acoustic streaming^83–85^. Acoustic radiation forces can be subcategorized into primary radiation forces that directly act on particles and secondary radiation forces that induce particle–particle interactions^86^. Primary acoustic radiation forces can move particles to pressure nodes or antinodes in a standing wave field^73^. Secondary acoustic radiation forces can be used to aggregate particles or to form assemblies by taking advantage of the inter-particle forces. A detailed theoretical analysis of acoustic radiation forces for various cases can be found in the works of Doinikov^85^ and Bruus et al.^87,88^.

The primary acoustic radiation force, ***F***_*R*_, acting on a compressible spherical object in a standing wave field is given by^89^,

1 $${\boldsymbol{F}}_{\boldsymbol{R}} = - \left( {\frac{{\mathrm{\pi} {p_o^2}V_{p}\beta _f}}{2\lambda }} \right)\phi \left( {\beta ,\rho } \right)\sin \left( {\frac{4{\mathrm{\pi}} {\boldsymbol{x}}}{\lambda }} \right)$$

2 $$\phi \left( {\beta ,\rho } \right) = \frac{{5\rho _p - \rho _f}}{{2\rho _p + \rho _f}} - \frac{{\beta _p}}{{\beta _f}}$$

where *p*_*o*_and *V*_*p*_ are the acoustic pressure and the volume of the particle; *β*_*f*_, *ρ*_*f*_, *β*_*p*_, and *ρ*_*p*_ are the compressibility and density associated with the fluid and the particle, respectively; and *ϕ*, *λ*, and ***x*** are the acoustic contrast factor, wavelength of the acoustic waves, and distance from a pressure node, respectively. Positive and negative acoustic contrast factors determine whether the force will be directed towards pressure nodes or antinodes, respectively (Fig. 2e). Particles and cells with different volume, density, or compressibility values experience varying magnitudes of acoustic radiation forces that affect their migration time and final position within and after the acoustic field.

Traveling acoustic waves can also induce an acoustic radiation force on suspended particles due to anisotropic scattering of waves that does not rely on the establishment of pressure nodes and antinodes. Skowronek et al. introduced a dimensionless coefficient *κ* = $\frac{2{\mathrm{\pi}} r}{\mathrm{\lambda}}$ to describe the effective acoustic radiation force for the manipulation of particles via traveling acoustic waves, where *λ* and *r* are the wavelength of acoustic waves in a liquid medium and the radius of the solid particles, respectively^90^. If *κ* < 1, then no net acoustic radiation force is applied to the particles, as the wave scattering is isotropic. If *κ* ≥ 1, a net acoustic radiation force drives the movement of particles in the fluid flow. Based on the elastic theory developed by Hasegawa et al.^91^, Destgeer et al. named the dimensionless number *κ* as “acoustic radiation force factor”^76^ since it described the acoustic radiation force per unit acoustic energy density per unit cross sectional area of a spherical object. They used this parameter to predict the frequency and particle size dependence for size-selective particle manipulation in a traveling acoustic wave field^76,79^. Based on these considerations, for successful traveling acoustic wave-based separation, the input frequency must be high enough with respect to the size of particles of interest^75,76^.

While acoustic radiation forces play a major role in manipulating particles, another important phenomenon leveraged in the acoustic separation is acoustic streaming, which arises from the viscous attenuation in a liquid and results in a net displacement of the suspended particles. Acoustic streaming can occur in various forms depending on the process and scale of the wave attenuation^92^. Details of various acoustic streaming mechanisms and their applications are discussed by Wiklund et al.^92^ and Sadhal^93^. Suspended inclusions experiencing acoustic streaming are subject to a drag force given by Stokes’ equation as^94^,

3 $${\boldsymbol{F}}_{\boldsymbol{d}} = 6{\mathrm{\pi}} \mu r{\boldsymbol{v}}$$

where *μ*, *r*, and *v* are dynamic viscosity of the liquid medium, radius of particles, and relative velocity of the particle with respect to the medium, respectively. The drag force and the acoustic radiation force are the two primary competing forces in traveling acoustic wave separation devices. The coefficient *κ* also characterize the dominant effect such that when *κ* < 1, the acoustic streaming is the dominant force in the system and suspended particles and cells follow the streaming flows.

## Applications of acoustofluidic separation

Acoustofluidic separation has been employed in a wide range of applications ranging from isolation of rare circulating biomarkers to differential focusing and separation of nanoparticles. A list of various acoustofluidic separation applications along with the separation characteristics is given in Table 3 to highlight the spectrum.

**Table 3:** Different applications of acoustofluidic separation

Separated samples	Flow rate	Recovery rate (%)	Purity (%)	Viability (%)
Blood cells from plasma1^100^	0.17 mL/min	95	98	–
Platelets from RBCs^19^	0.4 mL/min	99	–	–
Platelets from WBCs^102^	20 μL/min	98	–	98
WBCs from RBCs^105^	5 μL/min	88	54	–
Prostate cancer cells (DU145, PC3, and LNCaP) from WBCs^111^	70 μL/min	72.5–93.9	79.6–99.7	–
CTCs from WBCs^12^	20 μL/min	>83	–	90.4 ± 4.7
Live MCF-7, N2a, and hESCs from dead ones^114^	100 μL/min	49.7 ± 7.1	97.5 ± 2.5	–
Inflammatory cells from liquefied sputum^117^	10 μL/min	83.9 ± 5.1	–	87.1 ± 8.9
*Escherichia coli* from blood cells^121^	4.5 μL/min	–	95.65	–
Exosomes from whole blood^13^	4 μL/min	82.4	98.4	–
100 nm particles from 300 nm particles^132^	1.8 μL/min	86.3	–	–
Encapsulated cells from empty alginate beads^144^	8 μL/min	97	>98	85

## Separation of blood components

Separation of various blood components is valuable in diagnostics as abnormal amounts of each component can be indicative of various disease states. Alternatively, in therapeutic applications, transfusions of particular components can be used to correct deficiencies. The purity and viability of separated cells is critical for diagnostic accuracy and therapeutic efficacy. The major components of blood are red blood cells (RBCs, 6–8 µm in diameter), white blood cells (WBCs, 12–15 µm in diameter), platelets (1–5 µm in diameter) and plasma. RBCs are the most abundant cell type in blood, with approximately 4–6 million cells per microliter^95^. There are about 4500 to 11,000 WBCs and 150,000 to 450,000 platelets per microliter of blood. The liquid part of blood, plasma, contains various types of proteins, antibodies, and molecules. Each of these blood components have their unique functions and can be used as targets for diagnostic and therapeutic purposes. Centrifugation is the conventional method used to separate blood components. By spinning blood under a typical 3000 × *g* centrifugation force, three fractions can be identified: a clear solution with a yellow color that refers to the plasma in the most upper phase, a buffy coat that contains WBCs and platelets in the middle thin layer, and RBCs at the bottom. Besides centrifugation, filtration is also used in some cases. However, the technology based on centrifugation or filtration is bulky and not easily amenable to point-of-care applications. In addition, they have limited efficiency and biocompatibility^96–99^.

**Fig. 3:**
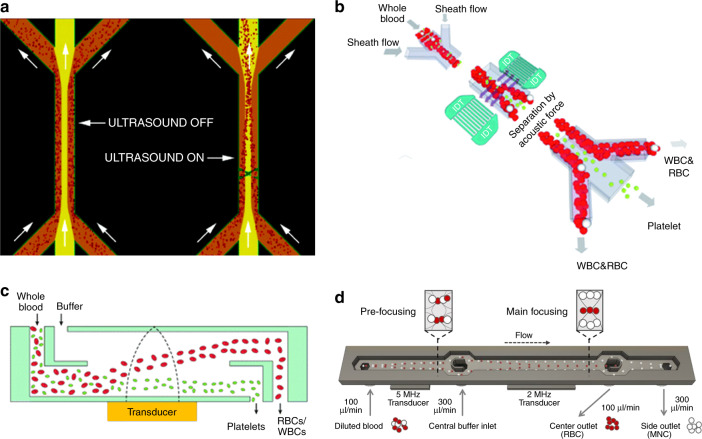
Acoustofluidic separation of blood components. **a** Separating blood cells from plasma by acoustic waves for the applications of blood wash or plasmapheresis^100^. Reprinted with permission from the American Chemical Society. **b** Separating platelets from blood cells by a SAW device^103^. Reprinted with permission from the Royal Society of Chemistry. **c** High-throughput separation of platelets and blood cells using a BAW technique^14^. Reprinted with permission from the Royal Society of Chemistry. **d** Separation of mononuclear cells (lymphocytes and monocytes) from blood using a two-stage acoustofluidic separation device^105^. Reprinted with permission from the Nature Publishing Group

Acoustofluidic separation technologies have been demonstrated with the ability to separate blood components in a continuous and biocompatible manner. In 2005, Petersson et al.^100^ reported the use of a BAW-based separation technology for the plasma exchange of blood, shown in Fig. 3a. This is desirable in certain applications where it is necessary to transfer RBCs from a carrier medium that contains high levels of inflammatory or coagulation factors. In a laminar flow microchannel, blood and clean plasma were injected through different inlets. The acoustic standing wave formed by the channel walls continuously translated blood cells from their original medium to clean plasma solution with virtually no mixing of the fluids. They achieved >95% recovery rate of RBCs and up to 98% removal rate of contaminants in a flow rate of 0.17 mL/min for the blood sample. This device can be applied for blood washing or plasmapheresis. Following this study, the same group demonstrated acoustic-based whole blood plasmapheresis^101^. RBCs were depleted from plasma to a level <6.0 × 10^6^ cells/mL, which fulfilled the benchmark requirements by the Council of Europe. The processing throughput was 20 μL/min (1.2 ml/h) for a whole blood sample. Though the efficiency matches the demands, the throughput needs substantial improvement in order to gain clinical significance. Adams et al.^68^ reported a temperature-controlled, BAW-based separation device in which a thermocouple was used to remove the heat generated by the transducer. In this case, they were able to use a high power intensity in order to boost the processing speed. The processing throughput was increased to 1 L/h with a RBC recovery rate of 95%.

Besides the separation of plasma from other components, acoustofluidic-based platelet separation has also been demonstrated by Petersson and his colleagues in 2007^19^. In this study, cesium chloride (CsCl) solution was added to tune the medium density. The combined effects of density and size difference enabled the separation of platelets from RBCs. They achieved 92% recovery of RBCs and 99% recovery of platelets. However, the addition of CsCl solution may raise health concerns for blood donors or patients. In order to be used for plateletpheresis, the side effects of CsCl need to be studied thoroughly. In 2011, Dykes et al.^102^ demonstrated the separation of platelets and WBCs without tuning the density of the medium. This technique was applied to remove unwanted, excess platelets for peripheral blood progenitor cell apheresis. The recovery efficiency of WBCs was 98% while 89% of platelets were depleted at a flow rate of 20 μL/min. By using a SAW-based separation device, Nam et al.^103^ achieved the separation of platelets from whole blood (Fig. 3b). The purity of platelets was close to 98% with a RBC depletion ratio over 99%. However, the processing throughput was only 0.25 μL/min, which needs to be increased for many real-world applications. In 2016, Chen et al.^14^ developed a high-throughput acoustofluidic separation device (Fig. 3c) that was demonstrated to separate platelets from whole blood at a flow rate of 10 mL/min. They achieved >85% platelet recovery rate and >80% RBC/WBC removal rate.

Recently, advancements have been made in separating RBCs and WBCs using acoustofluidics. In 2017, Kotz et al.^104^ reported the enrichment of lymphocytes with 55% recovery rate and 90% RBC depletion ratio by using BAW technique and tuning the diluent. Urbansky et al.^105^ reported that by changing the buffer conditions with different percentage of Stock Isotonic Percoll solution, the separation of WBCs and RBCs was successfully achieved in a two-stage acoustofluidic separation device, as shown in Fig. 3d. Lymphocytes and monocytes, two sub-types of WBCs, were separated from RBCs with a 2800-fold enrichment and 88% recovery rate. A throughput of 5 µl/min whole blood equivalent (>10^5^ cells/s) was achieved. Augustsson et al.^106^ demonstrated the separation of WBC subgroups via the difference in acoustic properties. They developed a method to form a suitable acoustic contrast gradient in the medium, thus subgroups of WBCs were focused to different positions where cells present zero acoustic contrast.

Acoustofluidic separation has also been used to separate lipid particles from blood as they carry the risk of clogging in blood circulation. Petersson et al.^70,107^ used standing acoustic waves to direct lipid particles to the pressure antinodes since lipids have a negative acoustic contrast factor. By using the half-wavelength acoustic resonator, cells were focused to pressure nodes located in the center of channel, while lipid particles were pushed to the side walls.

Acoustofluidic separation techniques have been demonstrated for a variety of blood component separation applications. The biocompatibility of these techniques has also been shown by some studies in terms of achieving low levels of platelet activation and preserving cell functions^108–110^. Although many advancements have been made over the past decade, there are still limitations of acoustofluidic-based blood component separation. For example, many of the techniques suffer from low throughput (in the µL/min range). The medical technology apheresis is well established and widely used as an FDA-approved treatment option for many diseases. Apheresis requires high-throughput (30–80 mL/min) processing of blood in a biocompatible manner, while simultaneously returning some components of the blood back into circulation. However, the current acoustofluidic separation techniques do not have the throughput needed for apheresis. In addition, some acoustofluidic techniques need to modify the carrier medium, which may cause issues when the blood components are returned to the body. In order for acoustofluidic techniques to find more clinical relevance, it will be important to increase the throughput and precision and avoid the use of undesirable carrier media.

## Separation of cancer cells

**Fig. 4:**
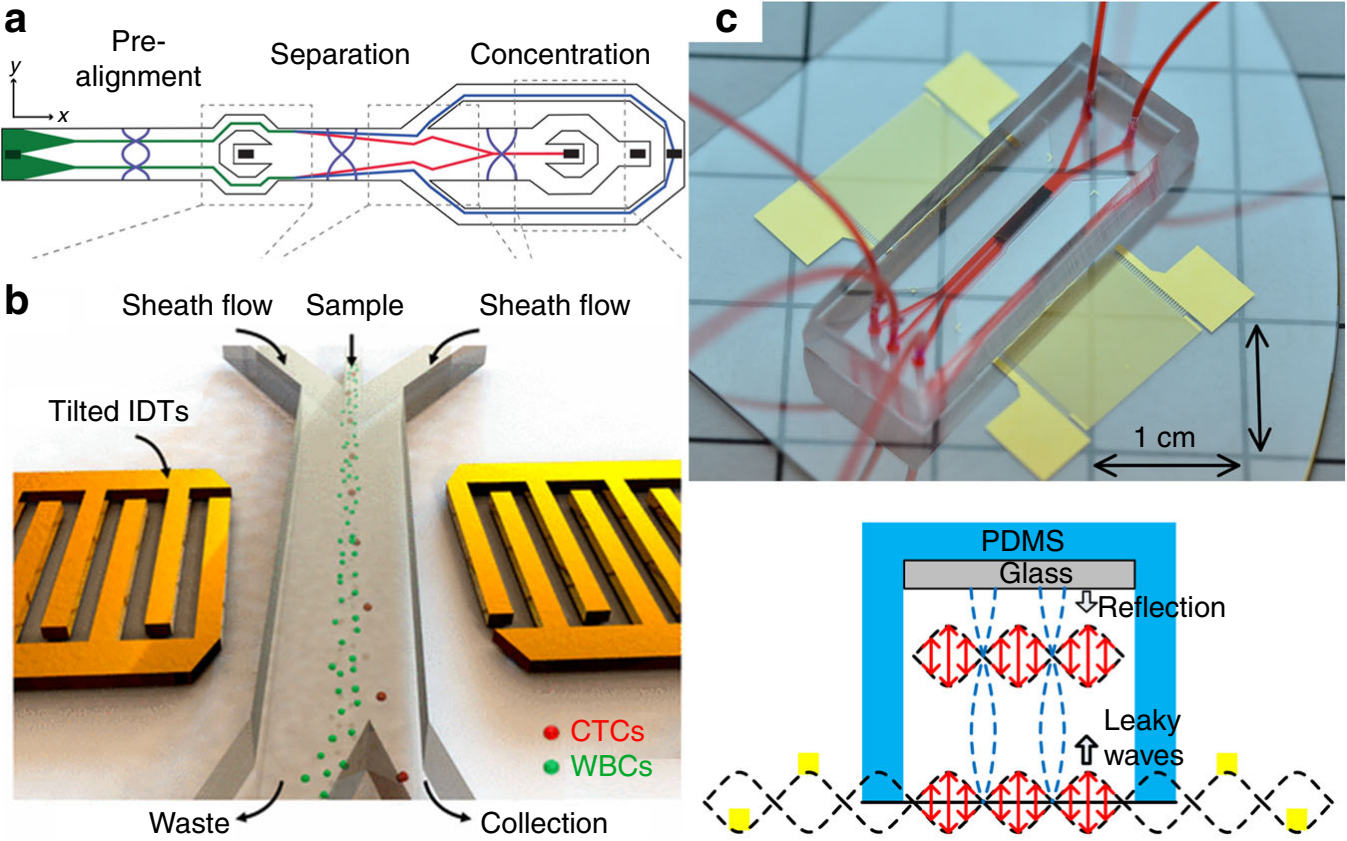
Acoustofluidic separation of cancer cells. **a** Separation of cancer cells using BAW techniques^69^. Reprinted with permission from the American Chemical Society. **b** Circulating tumor cells (CTCs) are separated from WBCs by using a tilted-angle SAW-based device^12^. Reprinted with permission from the National Academy of Sciences. **c** High-throughput isolation of CTCs by using standing SAW and PDMS/glass hybrid channel^55^. Reprinted with permission from the John Wiley & Sons, Inc

CTCs are cancer cells that leave the primary tumor, enter the circulatory system and can migrate to form secondary tumor cites. These cancer cells can provide valuable information for cancer diagnosis, help guide therapeutic interventions, and aid researchers in better understanding the mechanisms of cancer metastasis. To make use of CTCs, one needs to isolate them from peripheral blood in an efficient and rapid manner. In 2012, Augustsson et al.^111^ demonstrated the separation of cancer cells from WBCs by using a two-stage acoustofluidic separation platform which contained a pre-alignment module and a separation module. Cultured cancer cell lines were spiked into WBCs and isolated by acoustic field with 72.5–93.9% recovery rate and 79.6–99.7% purity at a throughput at 70 μL/min. Later an improved platform by Antfolk et al.^112^ achieved a recovery rate of 94.8 ± 2.8% of cancer cells with 2.2 ± 0.6% contamination of WBCs. In another work performed by the same group, a concentration module was added after separation, as shown in Fig. 4a (ref. ^69^). Magnusson et al. developed a clinical-scale automated acoustofluidic platform that can process 5 mL of erythrocyte-depleted paraformaldehyde fixed blood (diluted 1:2) at a flow rate of 75 μL/min, with 86 ± 2.3% recovery rate and 162-fold enrichment for breast cancer cell line cells^113^. Using the similar platform with Augustsson’s work, Zalis et al.^114^ also demonstrated the separation of live cancer cells from dead cells.

The previous studies demonstrated the separation of spiked cancer cells, but the situation is more challenging when processing patient samples since CTCs are extremely rare (typically 0–100 cells in 1 mL blood) in peripheral blood. Li et al.^12^ used a tilted-angle SAW technique and demonstrated successful isolation of CTCs from clinical patient samples, as shown in Fig. 4b. The processing speed for patient samples was 20 µL/min. To increase the processing speed, Wu et al.^55^ recently demonstrated a high-throughput (125 µL/min) acoustic platform for CTC separation. They systematically optimized the working conditions of the standing SAW-based separation platform, and modified the configuration of the channel, as shown in Fig. 4c. By inserting a piece of glass at the ceiling of fluidic channel, a vertical acoustic resonator was formed. Thus, the reflection of acoustic waves at fluid-glass interface significantly increases the acoustic pressure intensity which results in increased processing speed to enable practical applications.

## Cell filtering and cell washing

Acoustofluidic separation has long been used in perfusion culture applications before the concept of microfluidics. A seminal work in this area was published by Trampler et al.^115^. The mechanism relies on accumulating cells together using an acoustic radiation force. Suspension culture of mammalian cells was passed through an acoustic resonator where cells were trapped by the acoustic radiation force into parallel pressure nodes. After some time, gravitational forces begin to dominate and the aggregated cells sediment to the bottom of the reservoir, while the clarified medium is withdrawn and collected. The system retained up to 99% of the inflowing cells at a flow rate of 10 mL/min. Because of the demand of large volume, microfluidics was not suitable for perfusion in terms of mass production purposes. However, in certain applications requiring highly precise cell manipulations, microfluidic technology has been utilized. Thevoz et al.^116^ achieved synchronization of mammalian cells using acoustofluidic separation. Cells in different phases of development have significant differences in volume. Through size-selective acoustofluidic separation, label-free synchronization with 84% G_1_ phase synchrony was realized at a throughput of 3 × 10^6^ cells/h.

Acoustofluidic separation has also been implemented for cell washing, or separating cells from their original medium and replacing it with new carrier fluid. Li et al.^117^ demonstrated the use of tilted-angle SAW to transfer inflammatory cells from liquefied sputum samples. Cells were separated from the medium with residual dithiothreitol as buffer solution. Using a similar setup, Li et al.^118^ also effectively purified WBCs from cell debris. WBCs were transferred from lysed blood samples to PBS solution. By using BAW technology, Hawkes et al.^119^ and Petersson et al.^100^ performed translation of particles into clean medium from an original, contaminated one. The particles move toward the pressure nodes or antinodes under the standing acoustic wave. By locating the nodes in the clean medium, particles can effectively be transferred and washed. This technique has been implemented to wash off the fluorescein from a cell suspension and collect RBCs in clean blood plasma. In acoustic-based cell washing applications where particles are transported across a liquid–liquid interface, the properties of the medium need to be considered. It has been demonstrated that differences in acoustic impedances can cause unwanted relocation of liquids, resulting in decreased washing efficiency^120^. By altering the acoustic properties of one or both liquids, the relocation of liquids can be reduced and washing efficiency can be improved.

Apart from active removal of particles from contaminated samples, another strategy to achieve buffer medium exchange is to hold cells with acoustic waves while washing cells with a clean medium. Augustsson et al.^67^ used standing acoustic waves to confine cells to the center of the main channel. Buffer exchange was achieved via sequential cross-flow of particle-free buffer from branch channels.

## Bacteria separation

**Fig. 5:**
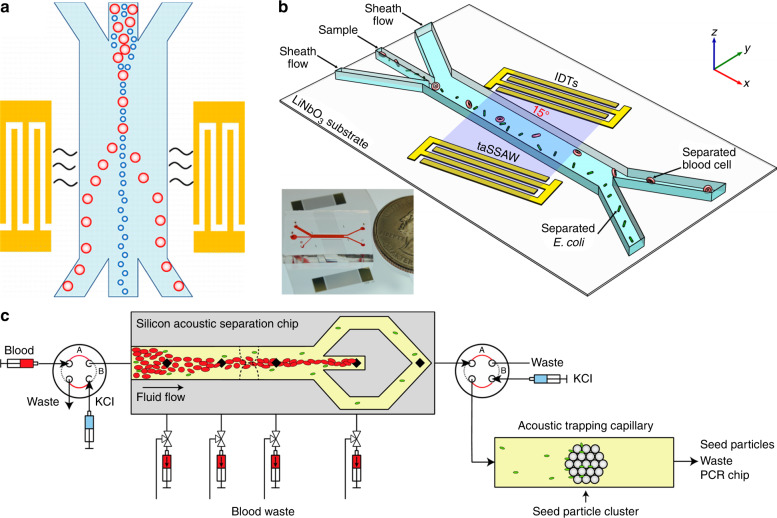
Acoustofluidic separation of bacteria. **a** Separation of bacteria from blood cells using a standing SAW technique^121^. Reprinted with permission from the American Chemical Society. **b** Blood cells are deflected by tilted-angle SAW field and thus separated from bacteria^122^. Reprinted with permission from the IOP Publishing. **c** Separation and enrichment of bacteria by acoustofluidics^123^. Reprinted with permission from the American Chemical Society

Separation of bacteria from specimens such as blood or sputum samples enables the identification of pathogens and sepsis diagnosis. In 2013, Ai et al.^121^ demonstrated the separation of *Escherichia coli* from blood cells by using a standing SAW technique (Fig. 5a). The bacteria was mixed with peripheral blood mononuclear cells and injected to the separation device. The sample was focused in the center of the channel initially by two sheath flows. The standing SAW formed two pressure nodes next to the side walls of the channel, thus peripheral blood mononuclear cells were driven to the pressure nodes and directed to the side outlets. The separation produced a sample containing *Escherichia coli* with 95.65% purity at a flow rate of 0.5 μL/min. Using a tilted-angle SAW separation technique (Fig. 5b), Li et al.^122^ demonstrated isolation of *Escherichia coli* from human blood cells with similar performance. As shown in Fig. 5c, Ohlsson et al.^123^ used a BAW technique for bacteria separation and enrichment. Firstly, a blood sample was processed in a half-wavelength resonator, where blood cells were focused at the center of the channel by the acoustic standing wave and sequentially separated from bacteria. The processing throughput was increased to 80 μL/min. Then, the cell-free bacteria sample was proceeded in a glass capillary. Beads were trapped in the capillary prior to bacteria sample by a localized acoustic standing wave field. The bacteria were attracted to the beads due to the primary and secondary acoustic radiation force. The acoustic trapping technique used here is also reported by another work published by Hammarström et al.^124^. Finally, by deactivating the acoustic field, the bacteria were released and collected for PCR based analysis.

Silva et al.^125^ reported the use of a disposable, plastic microchannel for acoustofluidic-based bacteria separation. The plastic device was built and tested by parametric rapid prototyping. After the optimization, the device achieved a 175% increase in throughput as well as reduced the power requirement by 82% relative to the baseline. Recently, Dow et al.^126^ used an acoustofluidic bacteria separation prior to detection. By depleting RBCs from the sample, the performance in a bacteriophage-based luminescence assay was significantly improved with a 33-fold increase in its detection limit.

Recently, Ohlsson et al. achieved acoustic separation of bacteria from blood cells at high cell concentrations by using impedance matched buffers^127^. High recovery rate (>90%) of the bacteria and high removal rate (>99%) of the blood cells was achieved for 5× diluted whole blood at a flow rate of 400 µL/min.

## Separation of nanoscale bioparticles

**Fig. 6:**
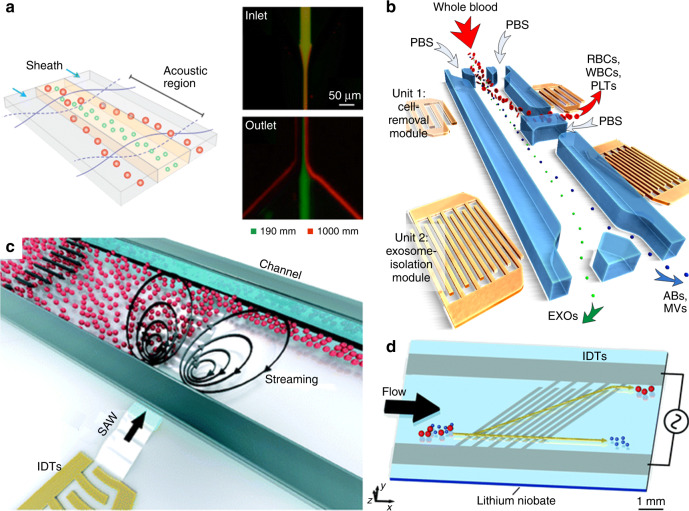
Acoustofluidic separation and manipulation of nanoparticles. **a** Purification of exosomes from microvesicles by standing SAW^15^. Reprinted with permission from the American Chemical Society. **b** Isolation of exosomes from whole blood by an integrated acoustic separation platform^13^. Reprinted with permission from the National Academy of Sciences. **c** Focusing nanoparticles using micro-vortex induced by high-frequency focused SAW^77^. Reprinted with permission from the Royal Society of Chemistry. **d** Separation of nanoparticles by integrating acoustic radiation force and dielectrophoresis^131^. Reprinted with permission from the Royal Society of Chemistry

Handling of sub-micrometer particles is challenging for acoustofluidics. As discussed in previous sections, acoustic streaming induced drag force becomes increasingly significant when the size of particles reaches the nanoscale. To overcome the streaming effect, one possible solution is to increase the frequency of the acoustic waves. BAW devices are typically operated in the frequency range of 100 kHz–10 MHz, thus it is difficult to deal with nanoscale particles^128^. On the other hand, the working frequency of SAW devices can be much higher (1 MHz–1 GHz)^129^. In this aspect, SAW-based devices show better potential in nanoscale separation. Lee et al.^15^ reported the separation of extracellular vesicles by using a standing SAW separation device working at 38.5 MHz, as shown in Fig. 6a. The cut-off size was set at 300 nm, so that exosomes were able to be isolated from other subgroups of extracellular vesicles. However, the processing throughput is limited at 0.43 μL/min. In 2017, Wu et al.^130^ demonstrated the separation of multiple types of nanoparticles by using tilted-angle SAW device. By using a single-phase unidirectional transducer which works at 33 MHz, the separation throughput can be significantly improved to up to 12 μL/min. The same group developed an integrated acoustic separation device for the isolation of exosomes from whole blood^13^. Two separation modules were combined within a single device: WBCs, RBCs, and platelets were removed in the first module and subgroups of extracellular vesicles were separated in the second module, as shown in Fig. 6b. The isolation of exosomes directly from whole blood was achieved with high purity and yield at a throughput of 4 μL/min. This integrated acoustofluidic device is the first platform that is able to isolate exosomes directly from whole blood samples. This acoustofluidic technique provides a potential point-of-care solution for exosome-based diagnosis.

While acoustic streaming is an obstacle for most acoustofluidic separation devices, it can also be harnessed to contribute to separation in some ways. Collins et al.^77^ developed a micro-vortex-based nanoparticle manipulation device via focused traveling SAW as show in Fig. 6c. Using focused IDTs and high-frequency (633 MHz) SAW, strong acoustic streaming was formed in the microfluidic channel. The streaming field focused fluid streamlines such that incoming nanoparticles were driven to a certain focal point regardless of their initial starting positions. Differential focusing of 100 nm, 300 nm, and 500 nm particles was achieved thus demonstrating the potential to separate nanoparticles of different sizes.

Another approach for nanoparticle separation is to integrate acoustofluidic technology with other mechanisms, such as dielectrophoresis. Collins et al.^131^ proposed to place IDTs underneath the microchannel so that electrodes were directly in contact with fluids (Fig. 6d). This way, nanoparticles experienced both an acoustic radiation force and a dielectrophoretic force. Using high-frequency SAW (50 MHz), successful separation of 500 nm and 300 nm particles was achieved. Recently, Sehgal and Kirby^132^ reported the use of a Fabry–Perot device which contained two Bragg reflectors to enhance the acoustic pressure. The separation of 300 nm and 100 nm particle was achieved in the Fabry–Perot system with 3-fold lower power density and 6.7-fold higher total flow rate compared to the conventional acoustofluidic system without the reflectors.

In addition to the previously mentioned methods, there is another unique technology known as acoustic trapping. With this technique, particles are trapped by the acoustic radiation force and their concentration builds at the acoustic pressure nodes. Once the concentration of particles trapped in the nodes reaches saturation, the trapped sample can be released and collected. As discussed before, the acoustic radiation force is very small for nanoscale objects; thus, trapping of sub-micron particles requires seeding micrometer-sized particles in the acoustic trap prior to the sub-micron particle capture. The pre-loaded micrometer-sized seed particles increase secondary acoustic forces so that they become significant. The secondary acoustic force is generated by the acoustic waves that are scattered from the micrometer-sized particles and it scales with the volume of both particles and the distance between them^133^. As such, the secondary radiation force generated from these particle–particle interactions enables successful trapping of sub-micron sized particles. Hammarström et al. demonstrated the enrichment of bacteria and particles down to 110 nm in diameter using a capillary and a 4 MHz transducer^124^. Evander et al. demonstrated the trapping of microvesicles using a similar approach^134^. Recently, Ku et al. successfully isolated exosomes and microvesicles from cell culture medium, human urine, and plasma samples^135^. The acoustic trapping method is mainly focused on enrichment, such that all the subgroups of nanoparticles or vesicles contained in the medium are simultaneously trapped with limited selectivity. However, it is a very useful approach when attempting to directly collect nanoparticles that are present at low concentrations in fluids.

## Separation of live cells from dead cells

Removing dead cells from a cell population is required for many biological and medical applications. Dead cells become permeable due to their compromised cell membranes that readily allow staining chemicals to enter the cytoplasm and interfere with downstream analysis^136^. In cell-based therapies such as stem cell transplantation, treatment potency of the cell therapy is reduced by high numbers of apoptotic cells^136^. Acoustofluidic separation is an ideal approach for removing dead cells from viable cells in a label-free manner. It does not involve steps that could alter the cell properties, such as labeling or exposure to different media. Yang et al. applied acoustic separation to remove dead mammalian cells from live cells using BAWs and enriched viable MCF-7 breast tumor cells from a mixture of dead and viable cells^137^. The separation mechanism is explained by the fact that dead cells, which experience a volume decrease after shrinkage in a high-salinity buffer, experience a lower magnitude acoustic radiation force. According to Equation 1, due to the difference in their volumes, residence times of dead and live MCF-7 cells become different and these two groups of cells are focused towards different outlets. At a flow rate of 12 mL/h, they reported the lowest contamination of viable cells. Later, Zalis et al.^114^ concentrated viable mouse neuroblastoma N2a and human embryonic stem cells using a two-stage acoustofluidic separation device. In the pre-alignment module, cells were focused to two lines by the standing BAW. Then, at the separation module, the second transducer generated a different frequency, thus forming a half-wavelength resonator. Live cells were relocated to the center of channel and separated from dead cells. They tested separation efficiencies for various live and dead cell samples obtained by different protocols and concluded that the acoustic separation can effectively isolate viable cells from dead cells regardless of their size difference.

## Droplet separation

**Fig. 7:**
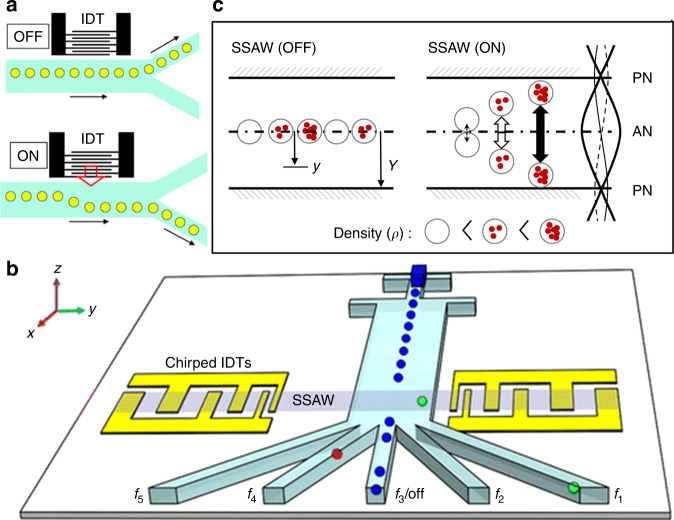
Acoustofluidic droplet separation. **a** Deflection of droplets by traveling SAW-induced acoustic streaming^141^. Reprinted with permission from the Royal Society of Chemistry. **b** Separation of droplets by changing the frequencies of slanted IDT^142^. Reprinted with permission from the American Society of Chemistry. **c** Separation of cell-encapsulated alginate beads based on density^144^. Reprinted with permission from the AIP Publishing

Droplet microfluidics is a powerful tool for applications such as drug discovery, cell incubation, and protein engineering, due to its advantages of low sample consumption, high throughput, flexible manipulation, elimination of cross contamination, and capacity to be integrated with other lab-on-a-chip devices^138,139^. In droplet microfluidics, droplets of interest often need to be isolated from the device. Wixforth et al.^140^ actuated small droplets on the planar surface of a piezoelectric chip using traveling SAW driven acoustic streaming, leading to programmable manipulation of droplets. Franke et al.^141^ used acoustic streaming to actuate the bulk fluid surrounding the droplets in a branched PDMS channel, as shown in Fig. 7a. After applying the SAW, droplets entering one outlet channel will be pushed to the other outlet. Standing SAW-based approaches are another promising way to separate droplets. Through the control of excitation frequencies, the spatial distribution of pressure nodes and antinodes will be changed and droplets under the corresponding acoustic radiation force can be easily and precisely sorted to different outlet channels (Fig. 7b)^142^. Vakarelski et al.^143^ used a similar standing SAW approach to separate a mixture of oil droplets and solid colloidal particles. The standing SAW-based approaches are particularly advantageous when high controllability and/or multi-channel separation are needed.

Besides water droplets in oil, the acoustofluidic separation technology was also used to manipulate cell-encapsulated polymer beads, e.g., hydrogel or alginate beads. Nam et al.^144^ reported the use of standing SAW to separate monodisperse encapsulated cells (Fig. 7c). Cells were randomly encapsulated in alginate beads, and the number of encapsulated cells determined the density of beads. The standing SAW separated beads containing multiple cells from those with small cell quantities or empty beads. They achieved a recovery rate of large-cell-quantity alginate beads up to 97% at a throughput of 2300 beads per minute.

## Acoustofluidic fluorescent-activated cell sorting

Acoustic technologies have been applied to perform the focusing and sorting function that is commonly found in fluorescent-activated cell sorting (FACS) systems^145,146^. One key parameter in the design of an acoustic-based cell sorting unit is the sorting throughput. In general, higher sorting throughput requires a minimum effective sorting distance which translates into a shorter processing time. If we assume the power input is not a limiting factor, the actuation time will solely rely on the effective acoustic field length, which can also be considered as the sorting resolution. Jakobsson et al. developed the first completely acoustic-actuated FACS system^147^. Two piezoelectric transducers are employed for particle focusing and sorting. Particles are first aligned to one side of the channel. When a target particle is detected, the BAW-based particle-sorting unit establishes a 1.7-mm-long standing acoustic wave field with a pressure node in the center of the channel. As a result, the target particle is deflected to the collection outlet with a throughput of 150 particles/s. This throughput is much lower than that of a commercial FACS, which typically can sort at a throughput greater than 10,000 particles/s. The reason for the low throughput of the BAW-based approach is that it is difficult to localize the acoustic field to a tiny region due to the dimensions of the BAW transducers.

**Fig. 8:**
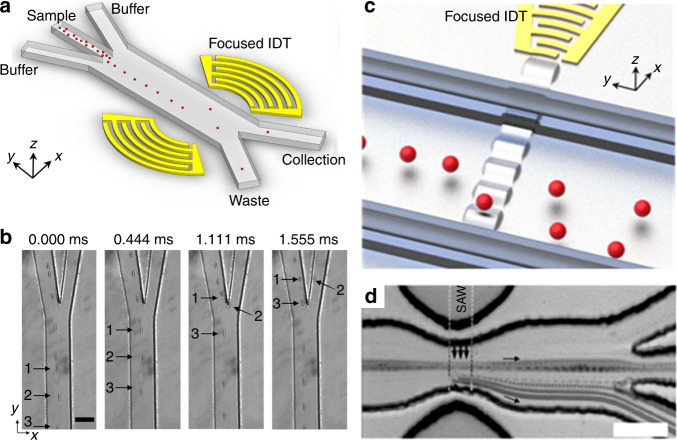
Acoustofluidic-based single particle/cell sorting. **a** A schematic of an acoustic sorting device using focused IDTs (not to scale). A sample solution with cells/particles to be sorted is infused from the middle inlet and a buffer solution is infused from both side inlets. **b** When a confined acoustic field is generated through focused IDTs, a polystyrene particle, labeled as 2, is pushed to the collection outlet from its initial path. Scale bar: 50 μm. **c** A schematic of an acoustic sorting device based on traveling SAWs using focused IDTs (not to scale). **d** A time-averaged image showing that 3 μm single particles are pushed to the lower outlet using 300 μs, 30 mW pulsed focused traveling SAWs with a beam width of 25 μm. Scale bar: 50 μm. Figures in **a**, **b** are adapted from ref. ^134^, and figures in **c**, **d** are adapted from ref. ^63^ with permission from the Royal Society of Chemistry

In contrast, it is easier to confine the length of the acoustic field with SAWs^148^. Therefore, SAW-based sorting units can achieve a much higher throughput^78,148^. Schmid et al. utilized traveling SAW-induced streaming for droplet and cell sorting^56^. They confined the area of the sorting region by using a small PDMS post as a coupling media for the SAWs which reduced the acoustic streaming only to a small length of about 150 µm. They report a theoretical maximum sorting throughput of 3000 droplets/s. Ding et al. presented a standing SAW-based multi-channel cell sorting unit^150^. The device uses chirped IDTs which can be excited through a range of frequencies. As a result, the position of the pressure nodes can be changed by adjusting the input frequency. Five-channel cell sorting is demonstrated, and a droplet sorting throughput of ~200 events/s is reported. Recently, in order to further improve the throughput of standing SAW-based cell sorting, Ren et al. designed focused IDTs to replace the commonly used parallel IDTs, as shown in Fig. 8a, b^149^. The focused IDTs are shown to significantly shrink the length of the sorting region to ~160 µm. A theoretical maximum sorting throughput of ~13,000 events/s, and an actual sorting throughput of ~3000 events/s has been demonstrated for 10 µm polystyrene particles. Collins et al. further demonstrated single particle sorting using traveling SAWs at 386 MHz in a PDMS microchannel, as shown in Fig. 8c, d^78^. By reducing the beam size down to 25 µm, they achieved single particle manipulation in a continuous flow with a sorting rate of up to 10,000 events/s. Acoustic-based parallel flow cytometry is a promising method to overcome the current throughput limit of about 50,000 events/s, which can make acoustic FACS a more favorable method for rare cell analysis, collection, and subsequent downstream culturing^151^. Recently, Ren et al. proposed a sheathless FACS system by using a standing SAW-based cell focusing unit that can focus cells into a single file at a designated position^152^. Sorting of mammalian cells (HeLa) at a sorting purity of greater than 90% and a throughput of 2500 events/s was successfully achieved.

## Summary and prospective

This review aims to give a comprehensive view of the state-of-art of acoustofluidic technology for the separation of nanoparticles, cancer cells, bacteria, extracellular vesicles, blood components, droplets, and other particles. These platforms will potentially benefit biological research and clinical applications such as the diagnosis and therapeutics of cancer, placental health monitoring, and treatments of cardiovascular heart disease. Other applications, such as the isolating of bacteria from water, food, and biological samples including blood, urine, sputum, or stool, is of great significance to infectious disease diagnosis and control. While many improvements have been made to acoustofluidic separation technology over the past decade, there are still many limitations. Firstly, it is noted that acoustofluidic separation technology is mainly used to deal with microscale objects such as cells, platelets, and bacteria, while few breakthroughs have been achieved for separation of nanoparticles. Specifically, it remains challenging to manipulate sub-100 nm objects including certain lipids, vesicles, viruses, proteins, and other biomolecules. The separation limit needs to be expanded at the sub-100 nm scale to meet the urgent demand of research regarding extracellular vesicles and to enable applications such as viral filtration. Secondly, the separation throughput, especially for SAW-based techniques, is limited. The current processing speed may be sufficient for some applications, but there is a significant need for rapid separation in many cases. One potential approach for overcoming these limitations may be the application of acoustic metamaterials and phononic crystals which can further improve the spatial resolution of acoustofluidic separation methods, enabling the direct manipulation of sub-100 nm particles. Application of acoustic metamaterials and phononic crystals can significantly increase the precision of acoustofluidic separation technologies without increasing the frequency^153–155^.

In addition to technological improvements, acoustofluidic researchers should look to address challenges in new areas of study. Platelet-based diagnosis is a rapidly growing area of research. With minor modifications, acoustofluidic separation technology will be able to isolate platelets from whole blood samples in an acceptable throughput for diagnostic purposes. Acoustofluidics also hold potential in leukocyte separations, which are used for stem cell harvesting and organ transplants. Separation of leukocytes using acoustofluidic technology will require improved throughput and increased precision to deplete erythrocytes. In addition to diagnostics and therapeutics, there are many other important directions to pursue, including industrial-scale nanoparticle purification, viral filtration, and phenotyping of non-cellular particles, such as lipids.

Ever since its infancy, acoustofluidic separation technology has been regarded with great potential due to its advantages for label-free, biocompatible, and contactless separation. Thanks to efforts from researchers in engineering, biology, and medicine, acoustofluidic separation technology has demonstrated its power in a variety of biological and biomedical applications for research and laboratory use. However, efforts are still needed to turn laboratory techniques into clinical instruments and point-of-care devices. Prototyping developments have been started, but challenges remain in terms of system robustness and the integration of fluid control modules, electronic designs, and user interfaces. The future work for acoustofluidic separation technology must not only be focused on technological improvements to the resolution and throughput, but also on two other key aspects: firstly, developing products and apparatuses for the clinical use; secondly, employing acoustofluidic separation technology to solve new problems in biology and medicine. The simultaneous focus on product development and technological improvements will enable acoustofludic technologies to find real-world applications and have an impact on the field of translational medicine.
